# Isolation of 1-(3′,4′-Dihydroxyphenyl)-3-(2″,4″,6″-trihydroxyphenyl)-propan-2-ol from Grape Seed Extract and Evaluation of its Antioxidant and Antispasmodic Potential

**DOI:** 10.3390/molecules24132466

**Published:** 2019-07-04

**Authors:** Michał Gleńsk, William J. Hurst, Vitold B. Glinski, Marek Bednarski, Jan A. Gliński

**Affiliations:** 1 Department of Pharmacognosy and Herbal Medicines, Wroclaw Medical University, 50-556 Wrocław, Poland; 2 Gretna Scientific LLC, Mount Gretna, PA 17064, USA; 3 Planta Analytica LLC, New Milford, CT 06776, USA; 4 Department of Pharmacological Screening, Chair of Pharmacodynamics, Jagiellonian University Medical College, 30-688 Kraków, Poland

**Keywords:** 1-(3′,4′-dihydroxyphenyl)-3-(2″,4″,6″-trihydroxyphenyl)-propan-2-ol, 3,4-diHPP-2-ol, procyanidins, grape seed extract, phenols, antioxidant activity, antispasmodic effect, marker for grape seed extract

## Abstract

HPLC profiling of phenolics in grape seed extracts revealed a prominent peak of an unknown substance with concentrations up to 5.3%. Spectroscopic data allowed the identification of the compound **1** as 1-(3′,4′-dihydroxyphenyl)-3-(2″,4″,6″-trihydroxyphenyl)-propan-2-ol. **1** is known to be produced from catechin and epicatechin through anaerobic bacteria from human, as well as the rat, intestines. It was hypothesized that the marc remaining after expression of juice from grapes became infested during storage, resulting in the production of **1**. Because compound **1** is infrequently found in nature and has never been found in grape seeds, its presence may be considered a marker of an unwanted anaerobic bacterial process occurring during production. The antioxidant potential of **1** was determined by DPPH, ABTS, and FRAP (ferric reducing antioxidant power) assays and compared to the potential of the following compounds: phloroglucine, pyrogallol, gallic acid, catechin, and epicatechin. Furthermore, it was established that **1** significantly reduced guinea pig ileum contraction induced by histamine.

## 1. Introduction

Proanthocyanidins are present in a wide variety of plant-derived foods and beverages, such as apples, grapes, chocolate, peanuts, and wine. A great variety of health-related properties have been reported for proanthocyanidins, including antioxidant, antiviral, anti-inflammatory, insulin-like activity, and anti-tumor activity. Antioxidant and cardioprotective properties of proanthocyanidins have gained considerable attention recently [1,2,3]. Grape seed proanthocyanidins (GSPs) comprise of dimers, trimers, tetramers, and higher oligomers of catechins and/or epicatechins, as described previously by Chen et al. [4]. In addition to the variability related to the number of connected epicatechin/catechin units, a specific type of connectivity, and chain branching, proanthocyanidins may also contain ester groups of gallic acid. The B-type procyanidins present in GSE (grape seed extract) contain mainly the 4β → 8’ bond and occasionally the 4β → 6’ C–C bond. GSPs display a range of beneficial biological effects, including anti-carcinogenic, vasoprotective, antioxidant, and neuroprotective qualities [5,6,7,8].

Fundamental concerns in quality control arise from the increasing use and the complex chemical composition of GSE. As there are no clear standardization criteria for its quality, the composition of commercial GSE supplements is subject to large variation [7]. 

The bioavailability, as well as bioactivity, of dietary polyphenols, are dependent on the activity of the colonic microbiota that, in turn, is largely influenced by their degree of polymerization (DP). Microbial catabolism in the colon is especially important for absorbed monomeric flavan-3-ols that reach the colon through bile and for molecules with a DP > 2, which present poor absorption in the small intestine [9]. Recently, it was reported that after oral administration of [^14^C] labeled procyanidin B2, 63% of total radioactivity was excreted via urine, indicating that the majority of the compound was degraded by gut microbiota [10]. The catabolism of flavan-3-ol monomers and polymers starts with the reductive cleavage of the heterocyclic C-ring, producing intermediate metabolite 1-(3′,4′-dihydroxyphenyl)-3-(2″,4″,6″-trihydroxyphenyl)-propan-2-ol (**1**). Subsequently, two metabolites are formed from **1**: 5-(3′,4′-dihydroxyphenyl)-γ-valerolactone and 4-hydroxy-5-(3′,4′-dihydroxyphenyl)-valeric acid, which, after a series of decarboxylation, dehydroxylation, and oxidation reactions, result in a great variety of chemical structures corresponding to phenylpropionic, phenylacetic, hydroxybenzoic acids, and simple phenols with different hydroxylation patterns [3,11,12,13].

Although the health effects of the procyanidin microbial metabolites remain in many cases incompletely understood, anti-inflammatory and antiproliferative activities of metabolites have been reported. Further findings suggest that their residence time in the organism is sufficient to produce local or systemic health effects [14,15].

There are only a handful of reports documenting the presence of **1** or the presence of other structurally similar substances in plants. Rotundifolinol, quracol A and B, propterol I and II, and virolanols II are examples of compounds isolated from plant material possessing similar structures to **1** [16,17,18,19,20].

Because **1** has not been described as a native constituent of grape seeds, its presence at such high concentrations must be interpreted as an artifact, likely resulting from the production process. Furthermore, the literature contains no information about the antioxidant activity of **1**. Therefore, its antioxidant potential was determined by DPPH, ABTS, and FRAP (ferric reducing antioxidant power) assays and compared to the potential of other closely related phenols, including phloroglucine, pyrogallol, gallic acid, catechin, and epicatechin.

Several flavonoids have been shown to have concentration-dependent relaxant effects in smooth muscle. Among them, (+) catechin has been shown to have an antispasmodic effect in rodents [21]. Moreover, Quracols A and B [18] are structurally similar to **1** and showed smooth muscle relaxation properties; also, phloroglucine is a commercial muscle relaxant in France. Based on these considerations, we evaluated **1** in a smooth muscle relaxing test.

## 2. Results and Discussion

**Figure 1:**
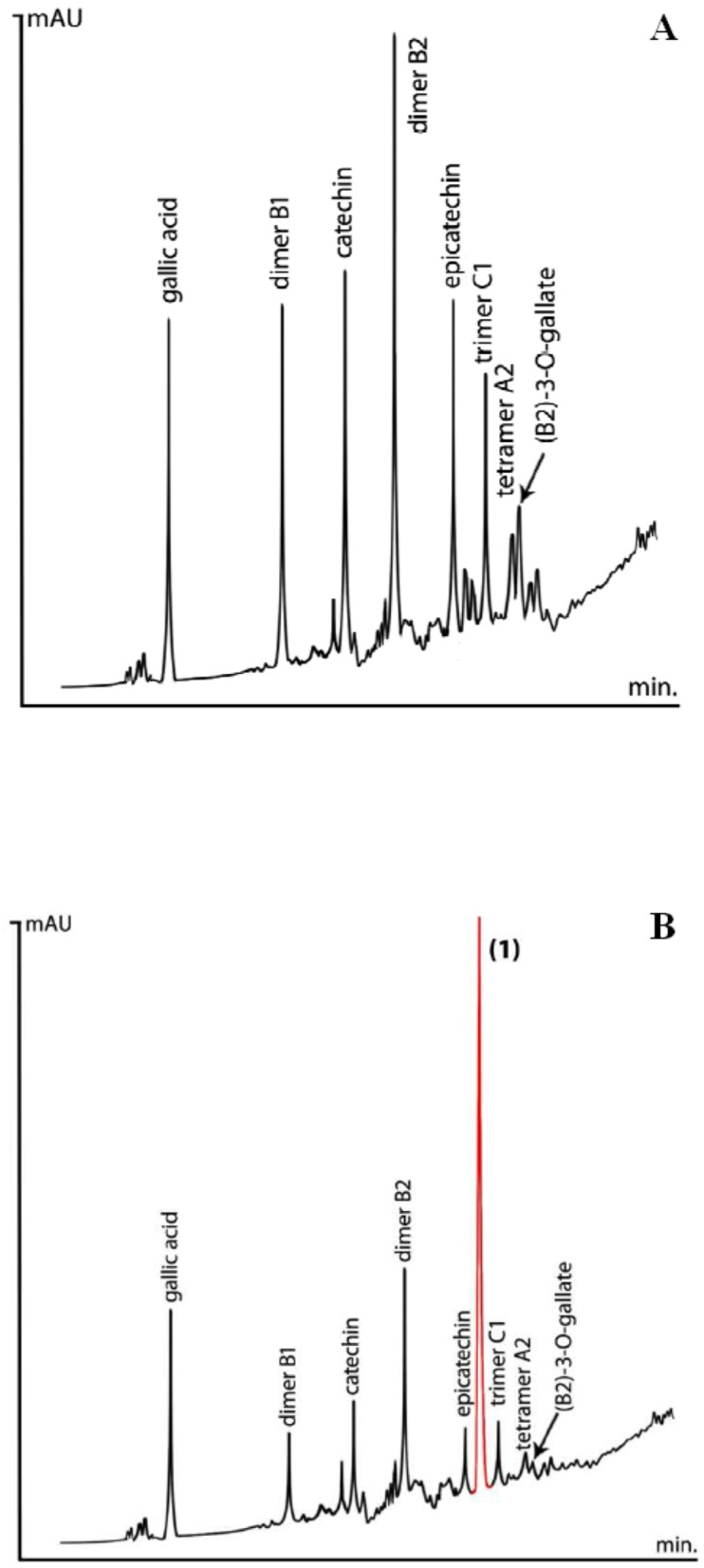
HPLC chromatograms. (**A**) Typical reverse-phase (RP) HPLC chromatogram of Grape Seed Extract, showing flavanols and major procyanidin components. (**B**) RP HPLC of Grape Seed Extract, containing 3,4-diHPP-2-ol (**1**).

In the present work, HPLC was performed on forty-six GSEs, acquired from domestic and international producers, to evaluate their procyanidin pattern. The main peaks typically distinguished in grape seed extract by reverse-phase (RP) HPLC belong to epicatechin, dimeric procyanidin B2, catechin, as well as several other dimeric, trimeric, and tetrameric procyanidins accompanied by their galloylated derivatives (Figure 1A). Observing procyanidins higher than DP 4 was difficult, considering peak overlapping, a complex baseline, and their relatively low natural concentrations.

In six samples, an unknown phenolic substance was observed, reaching 5.3% at its maximum (Figure 1B). The chromatograms (Figure 1A,B) revealed an inverse relationship between the peak area of **1** and the peak area of catechin, epicatechin, and procyanidins B2 and C1.

**Figure 2:**
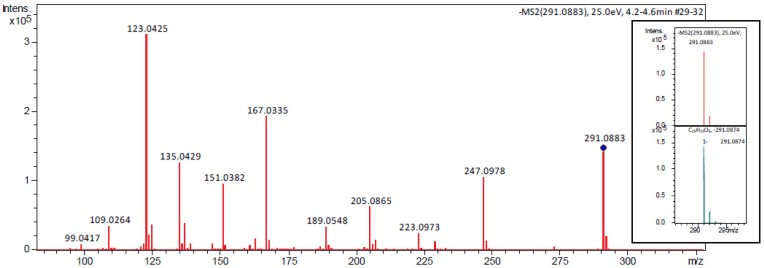
MS/MS spectrum of the (**1**), ion (*m*/*z* 291) in negative mode.

Following chromatographic purification and spectral analysis using NMR, MS, and MS/MS experiments (Figure 2), the peak’s identity was attributed to 1-(3′,4′-dihydroxyphenyl)-3-(2″,4″,6″-trihydroxyphenyl)-propan-2-ol (1). Both the NMR and MS spectra of 1 were in agreement with those previously published by Sánchez-Patán et al. [9], Meselhy et al. [22], Wang et al. [23], and Takagaki et al. [24].

So far, the presence of this compound originating from a plant source has been confirmed in one instance [16]. A few more reports exist on the presence of similar diarylpropane-2-ols of plant origin [17,18,19,20].

**Figure 3:**
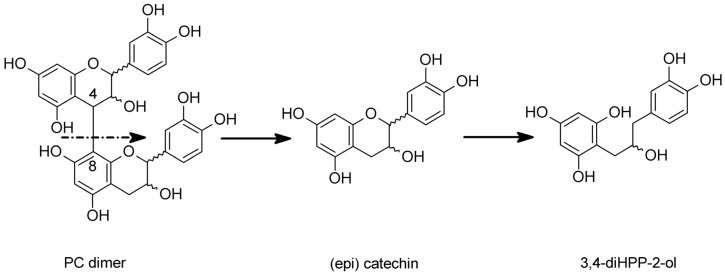
Tentative pathway for microbial degradation of procyanidin dimers, leading to the formation of 1-(3′,4′-dihydroxyphenyl)-3-(2″,4″,6″-trihydroxyphenyl)-propan-2-ol (**1**).

According to some authors, this compound is linked to the bacterial catabolism of monomeric flavan-3-ols ((+)-catechin, (−)-epicatechin, and (−)-epicatechin-3-O-gallate) [3,14,22,23]. A tentative pathway for microbial degradation of procyanidin dimers, leading to the formation of **1,** is presented in Figure 3.

Many reports present that during incubation with anaerobic bacteria of fecal origin, (epi)catechin or its galloyl esters can be converted into **1** and its related analogs through enzymatic hydrogenation [9,11,14,22,23].

In one report, it was concluded that *Lactobacillus plantarum* IFPL935 facilitates a reductive cleavage in the C-ring of monomeric flavan-3-ols and their gallates, both in solutions containing pure compounds and in complex phenolic extracts, producing diphenylpropan-2-ol. However, the report states that A- and B-type procyanidins, as both individual compounds and in extracts, do not undergo the same cleavage, and, therefore, suggests that the resistance is not connected to the catabolism under the conditions used in this study [9]. According to literature, besides *Lactobacillus plantarum* IFPL935, both Eubacterium SDG-2 and *Eggerthella lenta* rK3 isolated from human feces are the only bacteria capable of producing diphenylpropan-2-ol from monomeric flavan-3-ols [23]. Also, studies performed in vitro by Takagaki et al. [24], and in vivo by Margalef et al. [25], show the biotransformation of GSPs by rat intestinal microbiota. Similarly, compound **1** is formed in the lahpet-so, a traditional post-fermented tea of Myanmar produced under anaerobic conditions, and in the Japanese post-fermented tea produced by anaerobic microbial fermentation of green tea [26]. 

Although health effects of microbial metabolites have not been fully elucidated, antioxidative, anti-inflammatory, antiproliferative, and antiplatelet aggregation properties have been described for some polyphenol metabolites [9,14,15].

Our study shows that the presence of 1-(3′,4′-dihydroxyphenyl)-3-(2″,4″,6″-trihydroxyphenyl)-propan-2-ol in GSE indicates the involvement of anaerobic bacteria during the production of GSE from the grapes. Consequently, the bacteria reduce relative concentrations of epicatechin and catechin in GSE, while they increase the concentration of **1**. Therefore, compound **1** can be treated as a suitable marker for the detection of unwanted bacterial processes.

ABTS, DPPH, and FRAP assays were performed to determine the antioxidant activity of **1** and various related phenols.

Table 1 presents the results of the antioxidant activity of gallic acid, pyrogallol, 1-(3′,4′-dihydroxyphenyl)-3-(2″,4″,6″-trihydroxyphenyl)-propan-2-ol, epicatechin, catechin, and phloroglucine together with Trolox that was used as a reference.

It was found that all tested phenols were able to scavenge the ABTS•+ radical cation and antioxidant activities measured at 6 minutes, expressed as IC_50_, which were relatively higher when compared to Trolox. The IC_50_ value of **1** was the highest among all tested compounds. Similarly, in the DPPH assay, the activities of phenols were higher compared to Trolox with the exception of phloroglucine. The activity of **1** was slightly higher than epicatechin and catechin, but lower compared to gallic acid and pyrogallol. In this assay, phloroglucine showed the weakest activity. 

**Figure 4:**
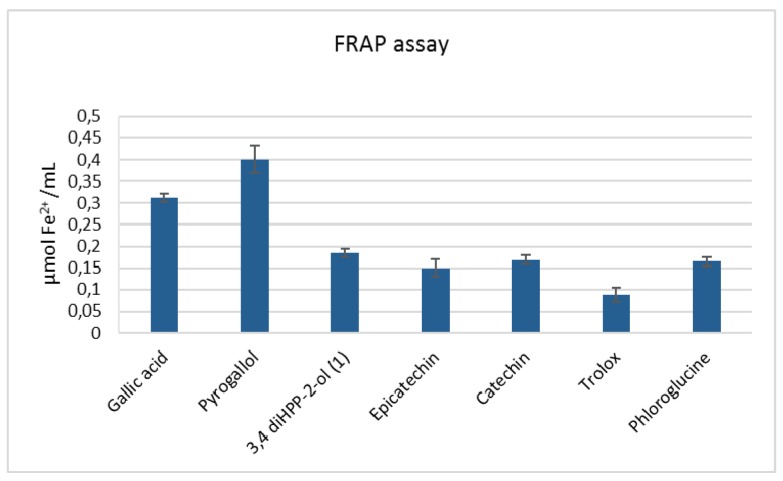
Antioxidant activity of various phenols determined via the FRAP (ferric reducing antioxidant power) assay. The concentration of the compounds was 0.01 mg/mL.

In the FRAP assay, all tested phenols possessed the ability to reduce Fe^3+^ to Fe^2+^, and their activity was higher than that of Trolox (Figure 4) at the same concentration. The antioxidant activity of phenolic substances in the FRAP assay depends on the number of hydroxyl groups. The antioxidant activity was higher for phenolic substances compared to Trolox, where only one hydroxyl group reacted in these experimental conditions.

**Figure 5:**
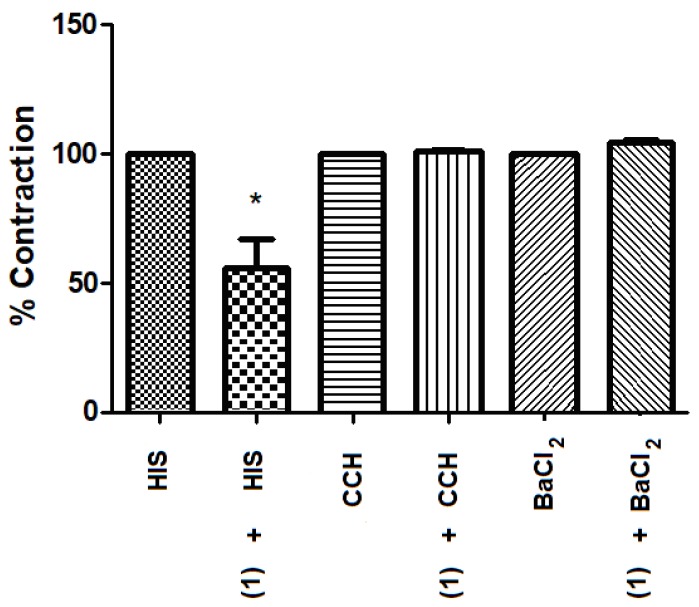
The spasmolytic effect of (**1**) 1-(3′,4′-dihydroxyphenyl)-3-(2″,4″,6″-trihydroxyphenyl)-propan-2-ol (10 μM) during contractions induced by histamine (HIS) at 2 μM, carbachol (CCH) at 100 μM, and barium chloride (BaCl2) at 0.03 M. Results are expressed as the mean ± SEM (*n* = 6), * *p* <0.05 vs. histamine control contractions.

During an in vitro smooth muscle relaxant assay for **1**, the contractions induced by histamine were significantly reduced by 55% in the presence of **1** at a concentration of 10 µM. It is known that histamine-induced contraction of intestinal smooth muscle via stimulation of H1-receptors. The results may suggest an antihistamine effect of the tested compound. The presence of the test compound did not significantly alter the contractions of the small intestine after administration of carbachol or barium chloride (Figure 5).

## 3. Materials and Methods

### 3.1. Chemical Reagents and Materials

Samples of commercial grape seed extracts (GSEs) were obtained from domestic and international producers. Vouchers of commercially available GSEs have been deposited in the Planta Analytica (New Milford, CT, USA) facilities. Gallic acid, (+)-catechin, and (−)-epicatechin were obtained from Extrasynthèse (Lyon, France). Pyrogallol and phloroglucine were obtained from Fluka (Steinheim, Germany). Analytical grade organic solvents and HPLC grade water were purchased from Pharmco AAPER (Brookfield, CT, USA).

### 3.2. Isolation of (**1**)

Ten grams of GSE was partitioned between 50 mL of water and 75 mL of ethyl acetate. Evaporation of the organic phase produced 3.7 g of a residue that was subjected to centrifugal partition chromatography on a Kromaton FCPC model A with a 1 L rotor. The solvent system consisted of ethyl acetate-ethanol-water (10:1:10, *v*/*v*). After equilibration by shaking, the lower phase was pumped into the rotor at 200 RPM. Next, the entire 3.7 g sample was dissolved in 30 mL of a 1:1 mixture of each phase and injected into the rotor, while the rotational speed was increased to 800 RPM. Following sample injection, the upper phase was pumped into the rotor at 9 mL/min. The eluent was collected into test tubes in 20 mL increments. The band of **1** eluted in test tubes #37–53. After combining and evaporating, this fraction produced 390 mg of **1** at 94% purity. The final purification was achieved by preparative HPLC using a Waters 600 system with manual injection (Rheodyne 7125) and a YMC ODS-AQ 50 × 250 mm preparative column. The chromatography was carried out with a gradient of 8–15% acetonitrile with 0.5% acetic acid at 30 mL/min. Half of the 390 mg sample of **1** was injected per run, and the main peak of **1** was collected. After evaporation and drying in vacuo, the total yield of **1** was 320 mg of a colorless solid with a purity of 99.7%.

### 3.3. Preparation of Samples for HPLC Analysis

Samples of GSEs were dissolved in 20% MeOH in water with a final concentration of 10 mg/mL, and the samples were filtered through a 0.45 μm membrane before injection.

### 3.4. HPLC Conditions

HPLC chromatographic separation was achieved using a Hewlett Packard HP Series 1050 (Agilent Technologies, Santa Clara, CA, USA) system equipped with an autosampler and DAD. A YMC-Pack C18, 4.6 × 150 mm, S-5 μm, 120 Å column was used for all analyses. The mobile phase was composed of acetonitrile and HPLC grade water. HPLC signals were monitored at 270 nm with a flow-rate of 1.0 mL/min. HP Chemstation A10.7 was used to control the operation of the system and perform data analysis. The injection volume of all solutions was 5 μL. The gradient elution system consisted of water with 5% acetonitrile and 0.1% trifluoroacetic acid (mobile phase A) and acetonitrile (mobile phase B). At a flow rate of 1.0 mL/min, the following elution program was used: 0 → 15.0 min (5 → 25% B), 15.0→15.1 min (25 → 90% B), 15.1 → 17.0 min (90% B), 17.0 → 17.1 min (5% B), 17.1 → 24.0 min (5% B). All analyses were carried out at 25 °C.

### 3.5. NMR, HRESIMS, and MS/MS Evaluation of **1**

NMR spectra were obtained on a 300 MHz spectrometer (Bruker BioSpin, Rheinstetten, Germany) operating at 300 MHz and 75.5 MHz, respectively, using standard pulse programs. Spectra were recorded in methanol-d4 at 295 K and referenced to solvent signals at 3.31 (δH) and 49.00 ppm (δC) (Figures S1 and S2).

The ESI-QTOF Compact (Bruker Daltonics, Bremen, Germany) was used for the HRESIMS and MS/MS experiments. The instrument was calibrated with the tuning ESI-L mixture (Agilent Technologies, Santa Clara, CA, USA). Analysis of the obtained mass spectra was carried out using Data Analysis v4.2 software (Bruker Daltonics, Bremen, Germany). The main instrumental parameters were as follows: scan range, 50–2200 *m*/*z*; dry gas, nitrogen; temperature, 180 °C; potential between the spray needle and the orifice, 4.2 kV. ESI was operated in negative mode. The HRESIMS spectrum showed an abundant [M−H]^−^ ion at *m*/*z* 291.0883 (calcd. 291.0874 for C_15_H_15_O_6_), indicating the molecular formula C_15_H_16_O_6_. The mass accuracy for **1** was within 4 ppm. In the MS/MS experiment, the negative ion [M−H]^−^ at *m*/*z* 291 was further fragmented at 25.0 eV, as shown in Figure 3.

### 3.6. Free-Radical Scavenging and Reducing Activity of **1** and Selected Phenols

All measurements were performed in triplicate using Spectrophotometer CE 3021 (CECIL instruments, Cambridge, Great Britain).

#### 3.6.1. ABTS Assay

Trolox™ (6-hydroxy-2,5,7,8-tetramethylchroman-2-carboxylic acid) (Sigma-Aldrich, Poznań,Poland) was used as the antioxidant standard. ABTS ((2,2’-azino-bis-(3-ethylbenzothiazoline-6-sulfonic acid) and potassium persulfate (dipotassium peroxydisulfate) were obtained from Sigma-Aldrich (Poznań, Poland). The Trolox solution was prepared in methanol at a final concentration of 1.76 mg/mL, then diluted with deionized water to form known dilutions ranging from 0.05 mg/mL to 0.44 mg/mL. Samples were dissolved in methanol at 1 mg/mL concentrations and then were further diluted five times with deionized water.

The assay was performed according to the method by Re et al. [27], with some modifications. The ABTS salt (7 mmol) and K_2_S_2_O_8_ (2.45 mmol) solutions were combined in a volumetric ratio of 1:1. The mixture was kept in the dark at room temperature for 16 h to produce the ABTS radical (ABTS^•+^). Immediately before the measurement, the mixture containing the (ABTS^•+^) was diluted with water to the absorbance of 0.7 at 734 nm.

Twenty microliters of each sample or the Trolox standard was added to the tube following the 2 mL of ABTS^•+^, and the resulting reaction mixture was vortexed. The absorbance was measured at 734 nm after a 6 min incubation at room temperature. Deionized water was used as a blank.

#### 3.6.2. DPPH Assay

A solution of 8.0 mg of DPPH (1,1-diphenyl-2-picrylhydrazyl; Sigma-Aldrich, Poznań, Poland) was dissolved in 20 mL of methanol. This solution was then stored in the dark for 2 h. Solutions of selected phenols were prepared in methanol at 1 mg/mL.

Measurements were performed according to Brand-Williams et al. [28], with some modifications. In six test-tubes, 0, 20, 40, 60, 80, and 100 µL of phenol samples were mixed with methanol up to a volume of 100 µL. Later, 2 mL of methanol and 0.25 mL of the prepared DPPH solution were added to each tube. The mixtures were vortexed and allowed to stand for 20 min in the dark at room temperature (25 °C). Absorbance was then measured at 517 nm. Methanol was used as a blank.

#### 3.6.3. FRAP Assay

FeSO_4_ × 7H_2_O was used for a calibration curve. The compound was dissolved in methanol and diluted with water to obtain concentrations of 0.0125, 0.025, 0.05, 0.10, 0.15, 0.20, and 0.30 mg/mL. From the phenol samples, 1 mg/mL methanolic solutions were prepared and diluted with water to finally obtain seven concentrations: 0.1, 0.08, 0.06, 0.04, 0.03, 0.02, 0.01 mg/mL, respectively.

The assay was performed according to Benzie and Strain [29], with some modifications. The FRAP working solution was prepared prior to the start of the analysis: 0.3 mol acetate buffer (pH 3.6), 0.01 mol TPTZ (2,4,6-tripyridyl-s-triazine; Sigma-Aldrich, Poznań, Poland) in 0.04 mol HCl (POCH, Lublin, Poland) and 0.02 M FeCl_3_ × 6H_2_O in water (iron (III) chloride hexahydrate; Chempur, Poland) were mixed in a volumetric ratio of 10:1:1 and protected from light. Next, 75 µL of the phenol solutions or FeSO_4_ × 7H_2_O solutions were mixed with 2.25 mL of the FRAP working solution and 225 µL of water. The obtained mixtures were incubated at 37 °C for 30 min, and their absorbance was measured at 593 nm. Deionized water with a FRAP solution was used as a blank.

### 3.7. Smooth Muscle Relaxant Assay

A 15 cm ileum segment was excised from the small intestine of male guinea pigs and immersed into a Krebs solution (NaCl 120 mM, KCl 5.6 mM, MgCl_2_ 2.2 mM, CaCl_2_ 2.4 mM, NaHCO_3_ 19 mM, glucose 10 mM). After the first 5 cm length closest to the ileocecal junction was discarded, 2 cm fragments were cut. Each segment of the ileum was placed in a 20 mL tissue organ bath system chamber (Tissue Organ Bath System—750 TOBS, DMT, Aarhus, Denmark) filled with the Krebs solution at 37 °C, pH 7.4, with constant oxygenation (O_2_/CO_2_, 19:1). The tissues were stretched by closing the clips between the metal rod and the force-displacement transducer. The preparations were allowed to stabilize in the organ baths for 60 min under a resting tension of 1 g and were washed every 15 min with fresh Krebs solution [30]. To evaluate the response and mechanism of action after the equilibration period, submaximal contraction induced by either histamine (2 μM), carbachol (100 μM), or BaCl_2_ (0.03 M) was determined in the presence or absence of the tested compound (10 μM) [21]. The Committee for the Animal Welfare of the Pharmaceutical Faculty of Jagiellonian University allowed usage of animal organs and tissues for the research (Document number 2018/09/01).

Contraction amplitudes were measured in at least six independent experiments. Relaxing responses induced by the tested compound were assessed as a percentage of the maximum contraction attained in control and expressed as the mean ± SEM. GraphPad Prism v5.0 software (GraphPad Software, San Diego, CA, USA) was used for data analysis, and the values were compared using the Student’s *t*-test for paired data.

## 4. Conclusions

GSEs are used as one of the most common antioxidants in many dietary supplements and herbal drugs. Processing and storage of grape seeds under non-GMP conditions may lead to contamination by anaerobic bacteria, resulting in the reductive cleavage of the C-ring of flavan-3-ols, thereby producing **1**. Our study indicates that **1** could serve as an important marker for the routine evaluation of the GSE quality. We established that **1** significantly reduced guinea pig ileum contractions induced by histamine, likely through blockage of the H1 histamine receptors. Consequences from the human intake of **1** on the relaxation of smooth muscles should be considered in the future. Additionally, as evidenced by the performed assays, we want to underscore the antioxidant potential of **1**, which exceeded the antioxidant potential of its parent molecules.

## Figures and Tables

**Table 1:** Comparison of pure phenolic compounds in terms of their radical scavenging activities.

Compounds	IC_50_ (µM) ^a^	
	ABTS Assay	DPPH Assay
Gallic acid	214.2 ± 8.1	12.03 ± 0.84
Pyrogallol	232.3 ± 15.9	15.36 ± 2.56
**1** 3,4-diHPP-2-ol	168.1 ± 9.7	17.67 ± 2.16
Epicatechin	254.3 ± 14.2	18.10 ± 2.88
Catechin	265.2 ± 10.5	22.21 ± 2.09
Phloroglucine	372.9 ± 45.6	207.51 ± 16.6
Trolox	921.5 ± 61.7	29.00 ± 2.37

^a^ Values are the means of three measurements (*n* = 3). [2,2’-azino-bis-(3-ethylbenzothiazoline-6-sulfonic acid)] free radical scavenging activity assay (ABTS), (1,1-diphenyl-2-picrylhydrazyl) free radical scavenging activity assay (DPPH).

## Supplementary Materials

The following are available online at https://www.mdpi.com/1420-3049/24/13/2466/s1, Figure S1: ^1^H-NMR spectrum of compound **1**; Figure S2: ^13^C-NMR spectrum of compound **1**.
