# Supplementary material for: Isolation of 1-(3′,4′-Dihydroxyphenyl)-3-(2″,4″,6″-trihydroxyphenyl)-propan-2-ol from Grape Seed Extract and Evaluation of its Antioxidant and Antispasmodic Potential
Source: Molecules. 2019 Jul 4;24(13):2466. doi: 10.3390/molecules24132466 (PMC6651082; doi:10.3390/molecules24132466)

Supplementary Materials

Isolation of 1-(3',4'-dihydroxyphenyl)-3-(2",4",6"-trihydroxyphenyl)-propan-2-ol from Grape Seed Extract and evaluation of its antioxidant and antispasmodic potential.

Michał Gleńsk [1,*], William J. Hurst [2], Vitold B. Glinski[3], Marek Bednarski[4], Jan A. Gliński[3]

[1] Department of Pharmacognosy, Wroclaw Medical University, Borowska 211a, 50-556 Wrocław, Poland
[2] Gretna Scientific LLC, 400 7th St., Mount Gretna, PA 17064, USA
[3] Planta Analytica, LLC, 461 Danbury Rd, New Milford, CT 06776, USA
[4] Department of Pharmacological Screening, Chair of Pharmacodynamics, Jagiellonian University Medical College, Medyczna 9, 30-688 Kraków, Poland
* Correspondence: michal.glensk@umed.wroc.pl; Tel.: +48-71-78-40-223

Figure S1: ^{1}H NMR spectrum of compound **1**

Figure S2: ^{13}C NMR spectrum of compound **1**

Figure S1: ^{1}H NMR spectrum of compound **1**

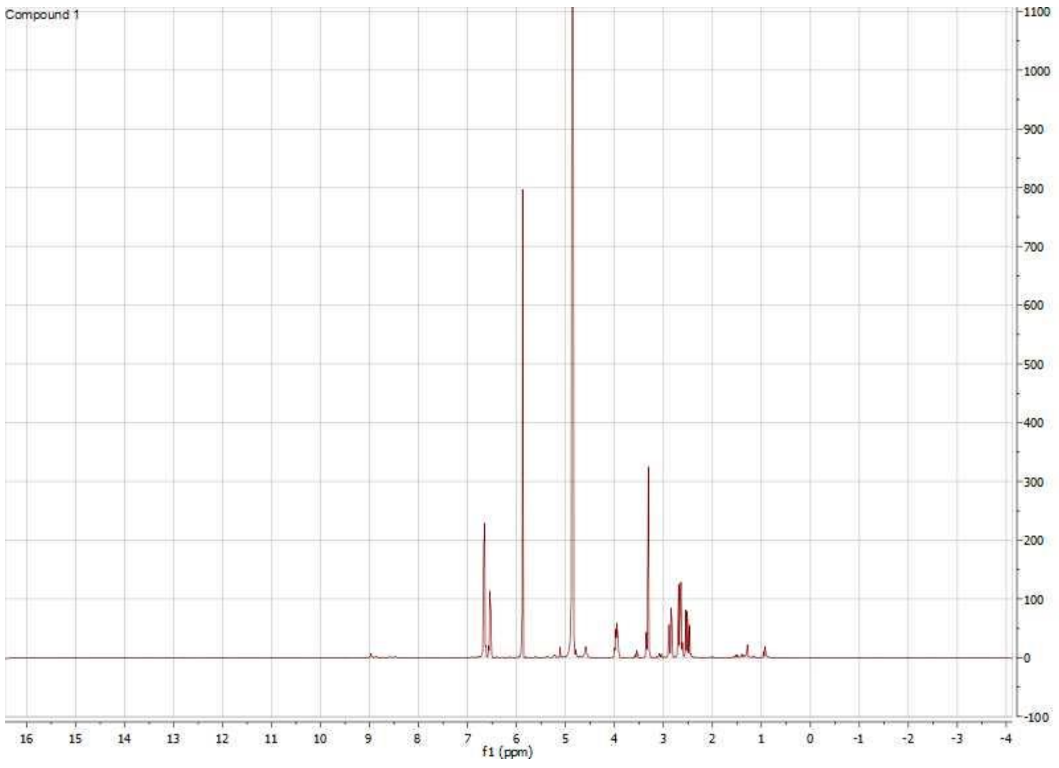

Figure S2: ^{13}C NMR spectrum of compound **1**

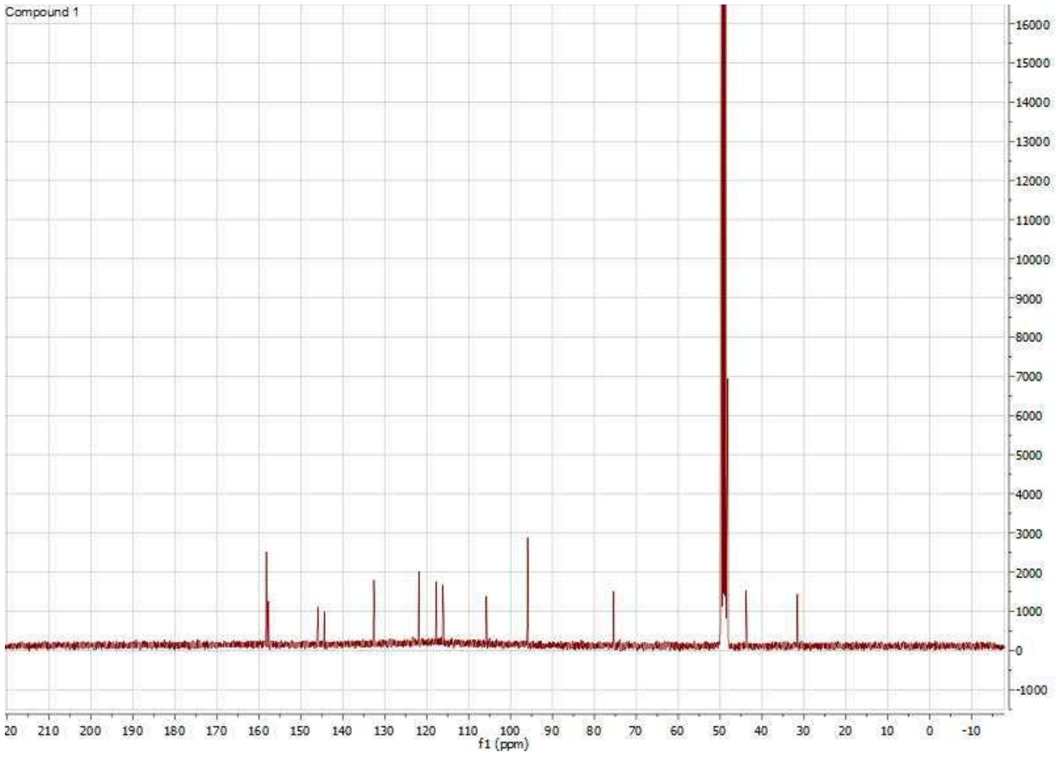

Supplement: Supplementary file 1 [file molecules-24-02466-s001.pdf]
